# Urea is a dynamic and functional component of mammalian breast milk

**DOI:** 10.64898/2026.09.28.755206

**Published:** 2026-10-12

**Authors:** Liisa Veerus, Haipeng Sun, Axel A. Almet, Erica R. Levin, Rachana Rao Battaje, Vibeke S. Dam, Dareen Shah, Rachel Clerk, Brandon Chang, Melissa A. Woortman, Joyce E. Stuckey, Margot J. Shumaker, Yue Sandra Yin, Adrija Kundu, Meliza Talaue-Weeast, Zhan Gao, Valeria Cardenas, Xue-Song Zhang, Donald D. Nyangahu, Heather B. Jaspan, Michael Manhart, Qing Nie, Emily Barrett, Lene N. Nejsum, Maria Gloria Dominguez-Bello, Martin J. Blaser, the New Jersey Kids Study team

## Abstract

Urea is a constituent of human milk, yet its regulation and interactions with the infant gut microbiome have not been established. Combining three human cohorts, a mouse model, and microbiome analyses, we show that milk urea increases across lactation, is conserved across mammals, and is not explained by maternal serum urea or diet. Instead, we identify lactation-dependent remodeling of mammary urea metabolism and transport, including dynamic epithelial AQP3 localization consistent with regulated urea transfer into milk. Alongside urea, maternal milk and stool provide reservoirs of urea-metabolizing bacteria that help establish the infant “ureabiome”, with encoded urea-utilization capacity. This maternal–infant axis is context-dependent: infant formulas provide higher urea levels than human milk, while functional assays show that urea supports both commensal and opportunistic bacteria in a concentration-dependent manner. Together, these findings define a maternal nitrogen axis linking milk urea to infant gut microbiome assembly and function.

